# Molecular Mechanism of Natural Food Antioxidants to Regulate ROS in Treating Cancer: A Review

**DOI:** 10.3390/antiox13020207

**Published:** 2024-02-06

**Authors:** Muchtaridi Muchtaridi, Farhah Az-Zahra, Hendris Wongso, Luthfi Utami Setyawati, Dhania Novitasari, Emmy Hainida Khairul Ikram

**Affiliations:** 1 Department of Pharmaceutical Analysis and Medicinal Chemistry, Faculty of Pharmacy, Universitas Padjadjaran, Sumedang 45363, Indonesia; farhah20001@mail.unpad.ac.id (F.A.-Z.); luthfi15002@mail.unpad.ac.id (L.U.S.); dhanianovita@gmail.com (D.N.); 2 Research Collaboration Centre for Radiopharmaceuticals Theranostic, National Research and Innovation Agency (BRIN), Jln. Raya Bandung Sumedang Km. 21, Jatinangor 45363, Indonesia; hend042@brin.go.id; 3 Research Center for Radioisotope, Radiopharmaceutical and Biodosimetry Technology, Research Organization for Nuclear Energy, National Research and Innovation Agency (BRIN), Jl. Puspiptek, Kota Tangerang 15314, Indonesia; 4 Integrated Nutrition Science and Therapy Research Group (INSPIRE), Faculty of Health Sciences, Universiti Teknologi MARA Cawangan Selangor, Kampus Puncak Alam, Bandar Puncak Alam 42300, Malaysia; emmy4546@uitm.edu.my

**Keywords:** cancer, molecular mechanism, natural food antioxidants, reactive oxygen species (ROS), oxidative stress

## Abstract

Cancer is the second-highest mortality rate disease worldwide, and it has been estimated that cancer will increase by up to 20 million cases yearly by 2030. There are various options of treatment for cancer, including surgery, radiotherapy, and chemotherapy. All of these options have damaging adverse effects that can reduce the patient’s quality of life. Cancer itself arises from a series of mutations in normal cells that generate the ability to divide uncontrollably. This cell mutation can happen as a result of DNA damage induced by the high concentration of ROS in normal cells. High levels of reactive oxygen species (ROS) can cause oxidative stress, which can initiate cancer cell proliferation. On the other hand, the cytotoxic effect from elevated ROS levels can be utilized as anticancer therapy. Some bioactive compounds from natural foods such as fruit, vegetables, herbs, honey, and many more have been identified as a promising source of natural antioxidants that can prevent oxidative stress by regulating the level of ROS in the body. In this review, we have highlighted and discussed the benefits of various natural antioxidant compounds from natural foods that can regulate reactive oxygen species through various pathways.

## 1. Introduction

According to the World Health Organization, globally, cancer is the second leading cause of death after cardiovascular disease. An estimated 13.4% of all deaths worldwide are caused by cancer [1]. The occurrence of cancer is caused by the division of malignant cells that are out of control, grow outside their normal limits, or spread to other parts of the body. In contrast to normal cells, cancer cells do not respond to control signals, which can cause cancer cells to grow and divide in an uncontrolled way and infect normal tissues and organs throughout the body. Cancer can be formed from a series of gene mutations that provide a cell with the ability to divide uncontrollably [2].

There are various types of cancer treatment, including surgery, radiotherapy, and chemotherapy. However, all of these current treatment options have shown a significant clinical challenge due to their damaging side effects and the possibility of provoking multidrug resistance. This cancer’s high death rate highlights the need for novel therapy approaches with fewer side effects. The difficulty in developing novel treatment lines with fewer side effects leads us to natural compound therapy. Natural compounds from natural foods offer several advantages, such as their ability to act on multi-molecular targets, minimal side effects, and convenience of access [3]. In order to target cancer cells, these natural compounds have a role as antioxidant agents that can regulate the levels of reactive oxygen species that can induce cancer cells’ progression. Several preclinical studies have revealed that certain dietary agents have promising roles in cancer therapy and prevention [4].

Reactive oxygen species (ROS) is a free radical produced during metabolism that has the ability to react with other molecules and has important roles in cell signaling, gene expression, and apoptosis [5]. It is associated with the regulation of the signaling processes of cell division, autophagy, inflammation, immune regulation, and stress-related responses. Uncontrolled production of ROS in cells can cause oxidative stress and cytotoxicity [6]. Oxidative stress is a condition caused by an imbalance in the production of reactive oxygen/nitrogen species (ROS/RNS) and antioxidant defenses. This condition has the ability to trigger cellular oxidative damage and is known as a major contributing factor in several diseases, from inflammation to cancer. The detailed molecular mechanism of the induction process of these diseases is still being studied, especially in certain aspects such as signaling pathways that associate excessive ROS levels, cell damage, and the initiation of cancer [7].

Antioxidants are the molecules that can neutralize free radicals by accepting or donating electron(s) to eliminate the radical condition [5]. These molecules can be either natural or synthetic compounds, but the synthetic one is not preferred due to its high possibility of causing adverse and toxic effects in the body [8]. Antioxidants have various pathways to target ROS and inhibit oxidative stress. By scavenging reactive oxygen species, preventing and repairing DNA damage, and modulating signal transduction pathways and gene expression, functional dietary components can enhance the natural antioxidant defense system [9].

Natural dietary components with antioxidant activity are mostly derived from phytochemical compounds. Plant-based foods for the diet are rich in antioxidant properties due to the thousands of bioactive antioxidant phytochemicals found [10]. In various botanical agents, there are many natural antioxidant agents such as polyphenols, terpenoids, vitamins, and so on [5]. Their antioxidant activity has promising potential to be used as an alternative therapy to treat various types of disease, including cancer. In this review, the pathway and molecular mechanisms to regulate ROS by natural antioxidants from natural foods are summarized, so it will be useful for researchers to conduct further research to find the further activity of those natural antioxidants and develop clear strategies by using functional food products as dietary interventions for cancer prevention and treatment.

## 2. The Search Criteria

### 2.1. Literature Search

**Figure 1:**
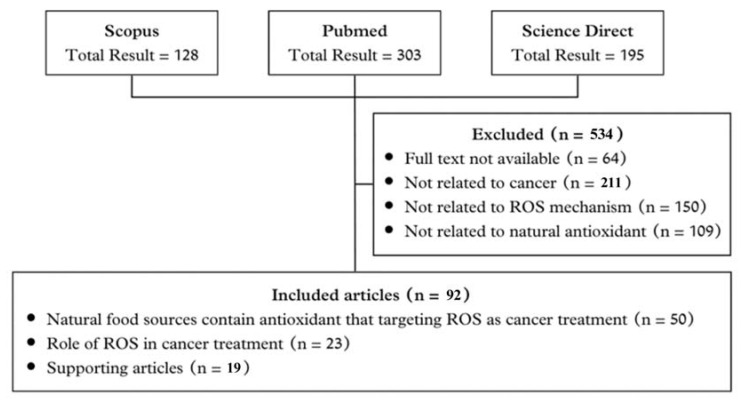
Literature search flow chart for search strategy and reasons for exclusion. ROS, reactive oxygen species.

In this review, the literature search aims to identify relevant articles that discuss the molecular mechanism of natural antioxidants from natural foods that can regulate ROS in order to treat cancer. We comprehensively selected electronic databases from PubMed and Scopus. Keywords that are included in this literature search are “molecular mechanism”, “ROS”, “cancer”, and “natural antioxidant”. The flow of the article search is shown in Figure 1.

We classified and excluded 534 articles that do not fulfill our required criteria out of 626 papers from various sources (Scopus 128; PubMed 303; Science Direct 195), in accordance with our search results mentioned in Figure 1. As a result, the total number of articles compiled in this article is 92.

### 2.2. Inclusion Criteria

Document type article was chosen, and the article must be written in English with a limitation on the publication year between 2005 and 2023, and its full paper is available. The selected articles were then evaluated based on several criteria, such as: (1) Antioxidant; (2) cancer cell; (3) cancer inhibition; (4) metabolism; (5) natural products; (6) oxidative stress; (7) reactive oxygen species; and (8) ROS. All articles must be related to the molecular mechanism of natural antioxidants coming from natural food sources that targeted ROS and showed as a potential candidate to treat cancer.

### 2.3. Exclusion Criteria

Articles not included in this review are in the form of book chapters, theses, dissertations, conference papers, articles that are not written in English, and articles that do not have related titles, abstracts, or keywords that are listed in the inclusion criteria. This study focused on the role of natural antioxidants as regulators of ROS in treating cancer.

## 3. Main Categories

### 3.1. Cancer and Oxidative Stress

Due to its multifactorial nature, cancer is the second-most lethal disease in the world, and its pathophysiology is still poorly understood. This makes cancer treatment difficult [11]. Cancer cells are cells that show abnormalities in normal cell cycle dynamics that result in the uncontrolled rapid division of malignant cells that do not respond to the controlling signal. Thus, it can grow and divide uncontrollably, infecting normal cells, tissues, and organs [9,12]. Clinically, cancer development may be initiated by internal factors (e.g., mutation of normal cells, immune conditions, or hormones) [13] and external factors (e.g., diet, tobacco or smoking behavior, environment, infections, or radiations) [14,15]. There are several oncogenic agents that can increase the risk of cancer cell proliferation. One of which is ROS, because it is associated with the initiation, progression, and metastasis of tumors. Nevertheless, this statement is still controversial due to the potential of ROS to be anticancer agents and crucial for the clearance of tumors [6].

ROS can be oxidizing agents that damage the DNA of the cells, resulting in malignancy and increasing the risk of cancer formation. The elevated levels of ROS cause oxidative stress, which is toxic to the body. Consequently, the body makes its own defensive system to keep the ROS levels at their safe threshold and prevent oxidative stress. This defensive mechanism is performed by antioxidant compounds. Balancing the level of ROS and antioxidants is important to maintain homeostasis and avoid cell damage [16]. An imbalance of ROS and antioxidant levels may cause oxidative stress that is related to the occurrence and development of various tumors, such as gastric cancer, colon cancer, and bladder cancer [17].

Antioxidants are available from a variety of sources. Our bodies have a natural defensive mechanism against oxidative stress in the form of endogenous antioxidants. An endogenous antioxidant defense system, including various antioxidant enzymes such as superoxide dismutase (SOD), catalase (CAT), glutathione reductase, and glucose-6-phosphate dehydrogenase, can act as components that maintain a cellular redox state by neutralizing free radicals. But in some circumstances, exogenous antioxidants are needed. Phytochemical compounds such as phenolic compounds and vitamins can provide this type of antioxidant property. All of these exogenous antioxidants may be necessary to maintain cellular redox and balance lipid, protein, and DNA oxidation [18].

In cancer cells, oxidative stress is a cycle that promotes carcinogenesis. ROS levels in cancer cells may increase and cause oxidative stress because of several factors, such as an increase in metabolic activity, dysfunction of mitochondria, an increase in cell signaling and peroxisome activity, and other oncogenes [16]. Since ROS can damage DNA and proteins within cells, oxidative stress is considered a serious condition that must be highlighted in preventing and treating cancer. For cells, oxidative stress is avoided due to its potential to damage cells, but intrinsic oxidative stress in cancer cells in malignant neoplasms may initiate cancer cell proliferation, genetic instability, and changes in cellular sensitivity to anticancer agents [19].

### 3.2. ROS Production in Biological System and Its Sources

ROS are inventible oxygen byproducts generated by the majority of physical and metabolic activity as a result of the oxidative phosphorylation process, which is mostly produced in mitochondria [5]. The formation of ROS occurs when molecular oxygen emits a single or more unpaired electrons, which, when in ground state, make this molecule not extremely reactive to other electrons, and the electrons in its outermost shell spin similarly. Once one of these two unpaired electrons becomes excited, it becomes extremely reactive, forming two electrons with opposing spins and whenever it reacts with other molecules, the oxygen will be reduced by one electron and become the stable intermediate, negative-charged super peroxide (O_2_^−^). This molecule is the major ROS precursor and constitutes an important mediator in oxidative chain reactions [16].

In a redox homeostasis state, the antioxidant defense system prevents the increased free radicals and reactive species that could potentially damage cells. In order to maintain the redox balance, cells produce several enzymes, including superoxide dismutase (SOD), catalase (CAT), and glutathione peroxidase (GPx), which are responsible for the first line of antioxidant mechanisms [20]. The SOD enzyme converts two O_2_¯ molecules into hydrogen peroxide (H_2_O_2_) and oxygen (O_2_) through a dismutation reaction. After anion superoxide is converted to H_2_O_2_, this molecule is either partially reduced to highly reactive hydroxyl radicals (OH*) in the presence of Fe or entirely reduced to water (H_2_O). In order to deflate the formation of OH*, CAT and/or GPX neutralize H_2_O_2_ to H_2_O and O_2_ even before OH* is created by the Fenton reaction [20]. Through these enzymes, the toxic superoxide radical and hydrogen peroxide are converted into harmless products and protect the cells from free radicals.

Since the chemical structures of ROS themselves contain unpaired electrons, these unstable molecules typically have a short lifespan and frequently depart from the location where they were produced within the cell after undergoing a reduction process. Among the various ROS, such as hydroxyl radicals or super oxide, hydrogen peroxide (H_2_O_2_) is often considered a stable and important signaling molecule in cellular processes with a longer half-life that allows it to diffuse across cellular membranes to regulate cellular signaling and oxidative stress. This condition also makes H_2_O_2_ suitable for long-distance signaling or sustained cellular responses [21].

Other radical molecules with a more unstable molecule and a shorter half-life, such as superoxide anion (O_2_^−^*) and hydroxyl radical (OH*), are highly reactive and typically involved in immediate cellular responses or damage. While these short-lived ROS are not typically considered stable signaling molecules such as hydrogen peroxide (H_2_O_2_), they can still play roles in cellular signaling through rapid and localized effects as they are able to initiate a chain reaction by removing a proton from non-radical biomolecules. Furthermore, these ROS can act as secondary messengers in specific cellular signaling cascades, such as those involved in stress adaptation, growth and development, and defense responses [22].

The level of ROS is determined by the rates at which they are produced as well as the existence and activity of cellular antioxidant defenses [23]. High levels of ROS can modulate cell signaling, dysfunction of mitochondria, an increase in peroxisome activity, and an increase in metabolic rates. High ROS levels can alter cell signaling, cause mitochondrial malfunction, enhance peroxisome activity, and accelerate metabolic rate. Cancer cells can employ this mechanism to generate cell cytotoxicity, and it can be exploited in medicine to develop novel therapeutic drugs that target cancer cells preferentially and selectively [16].

The ROS formation reactions were classified into three different categories. (1) ROS production as a metabolic side reaction; (2) ROS production as a response to pathogen attack; (3) ROS production as a response to abiotic stress [24]. In the metabolism process, mitochondria and peroxisomes are two organelles known as sources of ROS production. In mitochondria, ROS are generated by electron transport chain activity, and NADPH (nicotinamide adenine dinucleotide phosphate)-dependent oxidase is the primary generator of ROS production at the plasma membrane. On the inner mitochondrial membrane, there are complexes I–IV called electron transport chain (ETP) and an enzyme ATP synthase. ROS (O_2_^−^) are produced at complexes I and III, and most of them are leaked into the cytoplasm and dismutated by SOD (superoxide dismutase) to H_2_O_2_ [16]. Whereas in peroxisome, there is an enzyme known as named xanthine oxidase (XOD), one of the xanthine oxidoreductase (XOR) forms that generates H_2_O_2_ and O_2_^−^ through NADH oxidation [25]. ROS can also be produced to defend against pathogen attack. Recognition of pathogens in cells initiates ROS production and subsequently activates NADPH oxidase to spread hypersensitive responses. This response is generated to provoke the expression of genes and regulate secondary metabolism in order to protect cells from pathogens [24]. As a form of response to abiotic stresses from the environment such as drought, cold, heat, and salinity, more ROS will be produced, resulting in the over-accumulation of ROS, which has unfavorable consequences. However, this condition enables the body to establish an adaptation mechanism for abiotic stress. As a result, it becomes essential to regulate the level of ROS to remain within acceptable boundaries [26].

### 3.3. Regulation of ROS Levels

The level of ROS in cells should be balanced with the antioxidant levels to achieve the homeostasis that is needed for the survival of normal cells and suitable cell signaling. The production and elimination of ROS maintain an optimum level of ROS for pro-tumorigenic signaling. When the levels of ROS are decreased, the stimulation of signaling pathways to manage metabolic processes and cellular proliferation can be controlled. However, the generation and accumulation of ROS increase in cancer cells, creating hyperactivity in the cell signaling pathway for carcinogenesis. This can be due to a lack of tumor suppressors, hypoxia, oncogene activation, or increased metabolic activity. If the ROS level is too high, it will cause oxidative damage. Consequently, cancer cells maintain the accumulation of ROS by enhancing their antioxidant capacity. Enhancing the expression of antioxidants such as PRXs, SODs, CAT, GPx, GSH, and NADPH can scavenge the ROS to maintain homeostasis [6].

Numerous proteins, including Nrf2, NF-*κ*B, STAT3, and p53, play vital roles in cancer cell survival and resistance to drugs. Balancing these pathways for better chemotherapy results involves either blocking survival pathways or enhancing the cell death process. Therefore, both anti-apoptotic and pro-apoptotic effects resulting from modulating ROS levels can be used to regulate tumorigenicity and malignant progression [27].

Nrf2, or nuclear factor erythroid 2, is a transcriptional factor activated by oxidative stress that has an influential role in ROS regulation. In normal conditions, Nrf2 binds to Kelch-like ECH-associated protein 1 (Keap1), and in oxidative condition, the high levels of ROS may separate the Nrf2–Keap1 complex, resulting in activation of intracellular antioxidant activity, maintenance of cellular redox homeostasis, detoxification, and glutathione homeostasis. Nrf2 also governs the expression of ROS-eliminating enzymes and endogenous antioxidants. However, this process takes a relatively long time compared to the rapid production of ROS by active molecules, which bind to other proteins. The immediate effects of ROS interactions with cellular components are counteracted by the time-consuming activation of the Nrf2 pathway. This dual response system reflects the complexity of cellular defense mechanisms aimed at maintaining redox balance and preventing oxidative damage. Understanding these dynamics can guide research towards developing interventions that enhance the effectiveness of antioxidant defense mechanisms and reduce pathologies related to oxidative stress [18].

**Figure 2:**
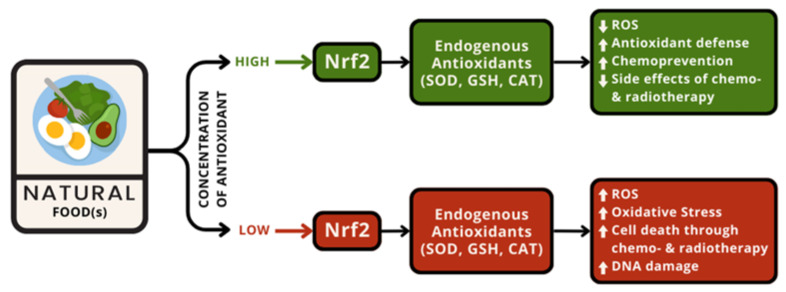
Role of antioxidant in natural food for cancer therapy is concentration-dependent. It is either enhancing or reducing ROS levels to achieve the opposing effects that both use for treating cancer.

The role of natural food as a natural antioxidant that specifically affects Nrf2 depends on its concentration, as mentioned in Figure 2. Different concentrations will induce different pathways related to ROS production.

Figure 2 shows that the Nrf2 modulation route may be separated into two parts: as a ROS inducer, which causes apoptosis in cancer cells, and as a ROS inhibitor, which can prevent broad cell damage, causing harm to other tissues [18].

NF-*κ*B or nuclear factor kappa B is a transcriptional factor that modulates gene expression related to innate immune response, cell proliferation, apoptosis, stress response, and organ development. It is normally localized in the cytoplasm, but when it is not complexed with Keap1 as a response to excessive oxidative stress, it can translocate into the nucleus and bind to ARE (antioxidant response element), resulting in the transcription of various antioxidants [28]. NF-*κ*B can be either a pro- or antioxidant agent, depending on the signaling and conditions in which it is activated. Activation of NF-*κ*B, which acts as an antioxidant, can suppress ROS levels, promote autophagy, inhibit JNK activation, and increase antioxidant targets. The mechanism of ROS suppression in this process may occur because increased NF-*κ*B activation leads to a decrease in the production of TNF-𝛼-induced ROS, which can trigger inflammation and cell apoptosis. Activation of NF-*κ*B signaling can also regulate autophagy by promoting autophagy, thereby influencing antioxidant defenses in the body. This condition can help remove oxidative proteins or damaged organelles, including mitochondria, which produce more ROS, thereby indirectly helping maintain redox balance [28,29]. The JNK (c-Jun N-terminal kinase) pathway is one of the proapoptotic regulatory agents, and its downregulation can serve an anti-apoptotic function [29]. ROS are known to cause prolonged activation of the JNK pathway. In particular, NF-κB activation can antagonize apoptosis induced by tumor necrosis factor (TNF)-α, a protective activity that involves suppression of the JNK cascade. A decrease in NF-*κ*B-mediated inhibition of JNK activation lead to increased TNF-alpha-induced apoptosis. Thus, blocking ROS may be an additional way for NF-*κ*B to inhibit JNK signaling. NF-*κ*B activation has also been shown to induce the transcription of antioxidant target genes including MnSOD (manganese superoxide dismutase), NADPH dehydrogenase [30] 1, heme oxygenase-1, and glutathione peroxidase-1. Conversely, activation of NF-*κ*B can also have a pro-oxidant role by inducing the transcription genes such as NADPH oxidase NOX2 subunit gp91phox [28,31].

STAT3, or signal transducer and activator of transcription 3, is a transcription factor that has a crucial role in various cellular activities such as cell growth, cell division, and the self-destruction of cells. This transcription factor is involved in the development and progression of many types of cancer. STAT3 can either act as a pro-oxidant agent by promoting ROS levels that contribute to cancer progression or as an antioxidant agent by upregulating the antioxidant defensive mechanism, which may protect cells from oxidative stress. Therapeutic implications of targeting the STAT3 pathway in cancer therapy are intriguing due to its potential to promote antitumor effects. A recent study reported a highly promising approach from targeted STAT3 for the treatment of CCA (cholangiocarcinoma). In this study, STAT3 inhibition by curcumin analog WZ26 showed that it could inhibit cancer cell growth and dreadfully induce ROS levels, leading to regulated mitochondrial apoptosis [32].

The p53 protein is an important tumor suppressor and a transcription factor that regulates a huge number of genes depending on cellular conditions. Since it is a common gene that is mutated in the majority of cancers, this protein is called “the guardian of cells or genome” [33]. When DNA damage occurs, the *TP53* gene (the gene that encodes the p53 protein) stops the cell cycle, and if the p53 protein is mutated, the cell cycle becomes unconstrained and the damaged DNA is replicated, resulting in uncontrolled cell proliferation and cancer tumors [34]. Most cancer cells with p53 mutations are less sensitive to chemotherapy than cells without p53 or with wild-type p53 [35]. However, there are several cases in which mutant p53 has little impact or increases cellular susceptibility to chemotherapy and radiation. The direct or indirect targeting of mutant p53 has been proposed as a possible cancer therapy method [36,37]. In order to become a good candidate for cancer therapy, the interplay between p53 and ROS regulation should be identified. This protein can act as a pro-oxidant when it induces the expression of genes involved in ROS production, leading to apoptosis. Conversely, p53 can also act as an antioxidant by regulating the encoding of genes such as SOD and glutathione peroxidase, which have antioxidant properties. The role of p53 is profoundly influenced by basal levels of ROS, whereas in normal conditions, p53 will promote antioxidant activity, and under severe oxidative stress, p53 will promote pro-oxidant activity by inducing ROS production [38].

### 3.4. Role of ROS in Anticancer Therapy

ROS play roles in various aspects of cancerous cell functions, including promoting their survival, facilitating angiogenesis, and aiding in metastasis. As a result, ROS are attributed to the initiation, development, invasion, and spread of cancer cells. Interestingly, even though ROS production is associated with these negative effects, it is also a fundamental mechanism utilized by many anticancer techniques such as chemotherapy, radiotherapy, and photodynamic therapy due to its connection to inducing cell death. Consequently, ROS are harnessed as agents to suppress tumors in the context of cancer treatment. This has led to ROS being referred to as a substance that has “double-edged sword” characteristics [16]. ROS have also been reported as a potential indicator to predict drug response after anticancer treatment [39].

Therapeutic approaches to treating cancer via regulation of ROS have two different mechanisms. Firstly, by reducing the amounts of reactive oxygen species (ROS) through the intake of antioxidants, which act as scavengers to hinder the ROS-signaling pathways and progression of cancer. One thing to keep in mind is that recent research indicates that the usual dosage of antioxidants may not be sufficient to counteract the high amount of ROS metabolites; thus, the current justification for using antioxidant supplementation during chemotherapy is to restore the total antioxidant loss caused by the depletion of antioxidant enzymes in cancer cells [40]. Another approach involves augmenting the formation of reactive oxygen species (ROS) to induce cancer cell death through senescence or apoptosis. Several chemotherapy treatments possess increased amounts of reactive oxygen species (ROS) within cells. This disrupts the balance of ROS in cancer cells, surpassing their harmful limit and triggering the activation of various pathways that lead to cell death [41]. Furthermore, exposure to anticancer drugs potentially triggers the oxidation of biomolecules or stimulates a mitochondrial-dependent response involving reactive oxygen species (ROS), significantly enhancing the lethal impact on cancer cells [41,42].

For instance, the anthracycline doxorubicin (DOX) produces hydroxyl radicals upon the reduction of semiquinone-DOX in mitochondria, leading to mitochondrial-mediated apoptosis. In addition, a recent study concluded that DOX targets the antioxidant enzyme peroxiredoxin 1 (PRDX1) in triple-negative breast cancer cells, causing mitochondrial-oxidative damage and activating the intrinsic apoptosis pathway [43]. In another case, the polyphenol curcumin shows a dual effect as an antioxidant to prevent tumorigenesis and a prooxidant agent to kill cancer cells. Curcumin induces the antioxidant enzyme activity to inhibit colon carcinogenesis in azoxymethane-dextran sulfate sodium (AOM-DSS)-treated mice [40,44] and reverses the glutathione levels in benzopyrene-induced mice [45], and this chemopreventive activity is also similarly demonstrated in curcumin analogs PGV-1 and CCA-1.1 to delay colon tumorigenesis in 1,2-dimethylhydrazine-induced rats. Conversely, curcumin and its analogs (PGV-1 or CCA-1.1) also bind to ROS-metabolizing enzymes (CBR1, NQO1, NQO2, GLO1, PRDX1, GTSP1, etc.) in cancer cells, which raises the intracellular ROS level and hampers the cell cycle progression, leading to cell death [46,47]. It has been noted that elevated ROS levels can trigger mitotic arrest in cancer cells. These findings suggest that the balance between prooxidants and antioxidants must be maintained for a functioning biological system, and most chemotherapy alters the redox equilibrium in cancer cells and normal ones and stimulates adaptive responses.

However, these two mechanisms still have their vulnerabilities. Promotion of tumor survival may occur when there is an intake of many antioxidant agents due to their tendency to inhibit ROS-derived apoptotic cell death. Conversely, promoting ROS production may cause normal cell mutagenesis, carcinogenesis, metastasis, and the upkeep of the phenotypical characteristics of cancer cells [16]. Cancer cells possess high levels of ROS compared to normal cells due to hypermetabolism, which supports the abnormal proliferation. The development of therapeutic approaches that promote oxidative stress by enhancing ROS capacity or blocking antioxidant processes has drawn a lot of interest lately. Increasing the accumulative ROS can potentially disturb redox homeostasis and eventually damage cancer cells through the apoptotic pathway [48]. Despite multiple studies demonstrating the efficacy of antioxidant medications in cancer treatments, no such evidence has been backed by large-scale, well-designed trials, which brings us full circle to the paradoxical nature of ROS behavior [49]. Indeed, numerous studies provide evidence that the suppression of antioxidant enzymes leads to the demise of cancer cells, particularly when this strategy is combined with therapies that enhance reactive oxygen species (ROS) levels. This approach offers an alternative to the typical method of focusing on oncogenes and tumor suppressor genes, which can be complex due to the large number of genes involved and their capacity to activate compensatory pathways [50].

### 3.5. Natural Antioxidants

**Figure 3:**
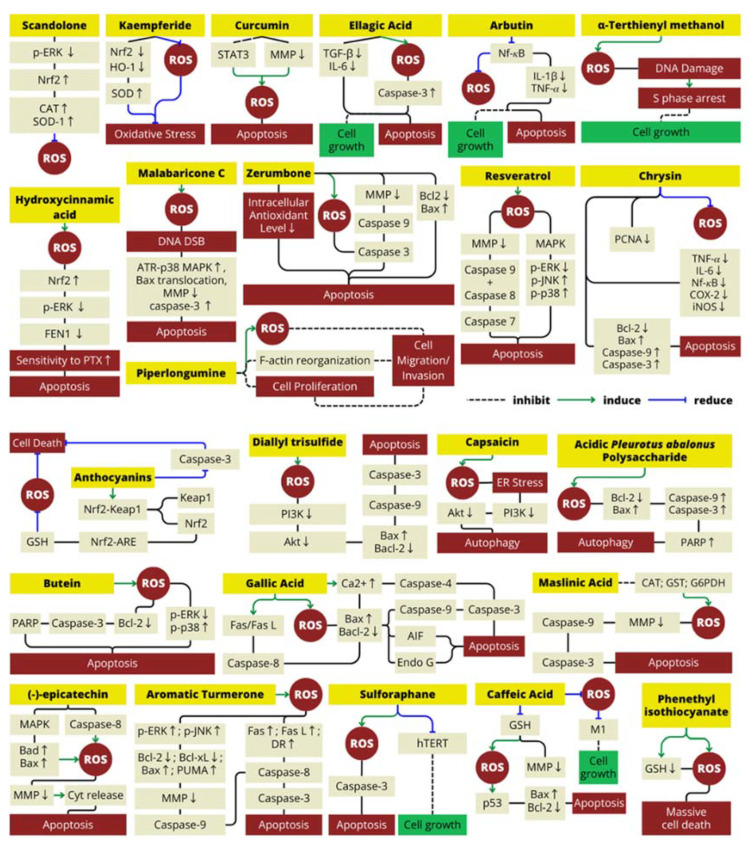
Mechanism of natural antioxidant compounds in ROS production.

Natural antioxidants are known to be widely distributed in natural food sources, such as plants, fungi, or even bee products such as honey or propolis. Several studies have demonstrated that these natural food sources have enormous potential as a source of natural antioxidants that may be utilized to prevent and treat a variety of harmful diseases, such as cancer, due to their biological activities. Polyphenols and their derivatives are one of the most common antioxidants contained in many parts of plants and exhibit a wide range of biological effects, such as anti-inflammatory, anti-aging and anticancer [51]. It has been reported that 25% to 48% of Food and Drug Administration (FDA)-approved anticancer agents are plant based in nature [52]. The list of natural foods that have activity as natural antioxidants and their mechanisms in ROS production have been summarized in Table 1 and Figure 3 below. 

Based on each mechanism mentioned in Table 1, all of these natural antioxidants found in the natural foods listed can act as either pro- or antioxidant agents by modulating ROS via various signaling proteins. It depends on its concentration, as explained in the previous paragraph. Figure 3 demonstrates the mechanism by which active antioxidant compounds control ROS production through changes in metabolic and signaling pathways, which can maintain oxidative homeostasis as well as defense mechanisms leading to cell death.

Polyphenols, carotenoids, and vitamins are the most common natural antioxidants derived from plant materials. Sources of these compounds can be found in fruits, vegetables, mushrooms, flowers, spices, etc. Polyphenol derivatives, hydroxycinnamic acids, are present in fruits, such as blueberries, kiwis, plums, cherries, and apples. Meanwhile, carotenoids are abundant in colorful edible plants, mostly in dark green and yellow-orange leafy plants. β-carotene commonly occurs in mango, pumpkin, carrot, nuts, and oil palm.

The isolation methods used to obtain these compounds depend on their solubility. Polyphenols and their derivatives, including phenolic acids, flavonoids, lignans, stilbenes and some vitamins, are mainly soluble in water (hydrosoluble antioxidants). Meanwhile, a group of natural pigments, carotenoids, and some other vitamins are soluble in lipids (lipid-soluble antioxidants). Consequently, the extraction procedure, as well as the solvent used to extract it, are critical for acquiring high concentrations of natural antioxidants [85].

Flavonoids are known to be sources of natural antioxidants, acting as reducing agents in several reactions. The mechanisms underlying the antioxidant effects of flavonoids include ROS scavenging, inhibiting oxidases in producing the superoxide anion, triggering the activity of antioxidant enzymes, and chelating trace metals. Owing to their chemical structures, the hydroxyl group on ring B and the 1,3-double bond conjugated with the 4-oxo moiety play crucial roles in reducing ROS, including peroxyl, hydroxyl, and peroxynitrite radicals [86]. It has been reported that quercetin, a flavonoid compound, could prevent ROS-induced oxidative stress and inflammation by suppressing NOX2 production. Another study revealed that rutin, a flavonoid glycoside, can increase ROS levels and promote the loss of mitochondrial membrane potential, thus activating apoptosis in CHME cells [87].

Derived natural compounds, such as polyphenols (including curcumin, quercetin, etc.), have demonstrated antioxidant activities that prevent oxidative damage in normal cells. On the other hand, they can also act as prooxidants, inducing higher intracellular oxidative stress and triggering cancer cell death. The hydroxyl group in polyphenols undergoes single electron transfer from hydroxyl or peroxyl radicals and decreases free radicals from ROS [88]. Furthermore, polyphenols inhibit the oxidase activators and enhance the ROS-metabolizing enzymes (peroxiredoxins, catalase, and superoxide dismutase) that are usually lower in tumor cells, thus eliminating excessive ROS levels and scavenging the redox equilibrium [89]. In addition, several studies have also reported that these polyphenols activate the transcription of antioxidant proteins such as peroxiredoxins and glutathione through NRF2 signaling [90]. Intriguingly, many antitumor polyphenols promote cellular growth arrest through the induction of a ROS-dependent premature senescence due to elevated intracellular ROS production and are considered a promising anticancer therapeutic strategy. Cellular senescence, once thought to be restricted to the cytoprotective mechanism of cells avoiding DNA damage from exogenous stress, is now considered a new target for anticancer therapy with senolytic and/or senostatic drugs [91].

Multiple investigations have also emphasized the ability of natural antioxidant chemicals to affect mitochondrial functions in addition to regulating the levels of ROS in cancer cells [91]. They can act both in the intrinsic and extrinsic pathways to regulate the ROS level and cause apoptosis. A study by Yun et al. revealed that vitamin C, as an antioxidant from dietary intake, increased the concentration of ROS levels to promote the death of colon cancer cells bearing KRAS and BRAF mutations [92]. However, another study showed that administration of N-acetylcysteine (NAC) and the vitamin E analog Trolox increased the invasive characteristics and migration of human malignant melanoma cells [93]. In an intrinsic pathway, these natural antioxidants will govern the mitochondrial membrane potential and trigger apoptosis. The mitochondrial membrane potential decreases due to the high level of ROS, leading to an imbalance between the pro-apoptotic (Bad and Bax) and the anti-apoptotic (Bcl-2 and Bcl-xl) proteins. This condition causes the transition in mitochondrial permeability [71]. Dissipation of mitochondrial membrane potential is the most-recognized apoptosis inducer that is triggered by the caspases, and it causes an increase in mitochondrial membrane permeability, the release of cytochrome oxidase C, and apoptosis. It also activates caspase-3 and caspase-9, enzymes that play pivotal roles in apoptotic cells [17]. Since mitochondria influence the induction of apoptosis, changes in mitochondrial membrane potential are frequently associated with cell death. Zerumbone from Zingiber zerumbet rhizomes has been examined and resulted in a significant increase in cellular ROS level, decreasing the cellular antioxidant level, and causing the loss of mitochondrial membrane potential [52]. Galangin and chrysin derived from honey can induce apoptosis through p38 mitogen-activated protein kinase (MAPK) and Bax activation, which causes the loss of mitochondrial membrane potential in B16-F1 and A375 melanoma cell lines [11]. Hence, their ability to selectively affect tumor cell behavior based on their differential redox status can be further explored as a rational target for adjuvant cancer therapy.

## 4. Conclusions and Recommendation

ROS imbalances are associated with numerous human diseases, including various forms of cancer. The interaction that occurs between ROS and natural antioxidants derived from natural foods demonstrates the significant potential of utilizing natural antioxidants in the development, prevention, and treatment of cancer. Antioxidants themselves play a pivotal role in regulating the levels of ROS in cells, either by maintaining them at a controlled level to prevent metastasis or by increasing their levels to induce apoptosis, which can kill cancer cells themselves. At the molecular level, ROS can influence cells through both intrinsic and extrinsic pathways to create the perfect circumstances for apoptosis.

From a literature search, it is evident that natural antioxidants play a crucial role in controlling the levels of ROS in the body and can be used in cancer therapy. Additionally, the main issue with cancer treatments to date is their poor clinical outcomes, which has led to alternative approaches, such as the use of natural antioxidant foods that exhibit low side effects. Consumption of specific natural foods, such as pomegranates and certain rhizomes such as turmeric and galangal, can aid in cancer healing due to their high natural antioxidant content. This might potentially become a reliable strategy to regulate ROS production in malignant cells. Nevertheless, further research is required to determine the appropriate dosage for consuming these natural foods to achieve optimal results with minimal side effects. Further studies are needed to assess the safety and efficacy of these potential natural sources and to determine the optimal dose for minimizing and identifying potential side effects.

## Figures and Tables

**Table 1:** The natural food antioxidants reported in literatures that can regulate ROS to treating cancer.

Natural Antioxidant	Type of Antioxidant	Food Source(s)	Molecular Mechanism	Cancer Line Cell(s)	Reference(s)
Scandolone	Flavonoid	*Cudrania tricuspidata* (Chinese mulberry)	Decrease ROS levels in cells by suppressing the protein levels of p-ERK that inhibit Nrf2 expression, increasing the expression levels of Nrf2/HO-1 and elevating the expression levels of CAT and SOD-1	LNCap and CRPC (prostate cancer cell line)	[53]
Kaempferide	Flavonoid	*Alpinia officinarum* (galangal rhizome) and *Hippophae rhamnoides* (sea-buckthorn’s fruit and seed)	Reduce oxidative stress by decreasing the levels of Nrf2 and HO-1 expression; increased the SOD content	HepG2 (human liver cancer cell line)	[54,55]
Curcumin	Polyphenol	*Curcuma longa* (turmeric rhizome)	Increase ROS (H_2_O_2_) production by reducing mitochondrial membrane potential and inhibiting the activation of STAT3 signaling that induce apoptosis and cell cycle arrest in cancer cells; promoted MAPK (mitogen-activated protein kinase) pathway activation	CCA (human cholangiocarcinoma cell line); human glioblastoma multiforme (GBM) cell line	[32,56,57]
Ellagic acid	Polyphenol	*Punica granatum* (pomegranate fruit)	Inhibit cell growth and induces apoptosis; increase ROS, activate caspase-3; reduce TGF-beta and IL-6 that inhibit cancel cell apoptosis	LNCap and PC3 (prostate cancer cell line)	[58,59,60]
Arbutin/hydroquinone-β-d-glucopyranoside	Polyphenol	*Arctostaphylos* sp. (bearberry)	Reduce ROS levels and decrease proliferative gene that characterized by an increase in apoptosis via reduction in the activation of NF-*κ*B that reduce the expression of downstream genes, including IL-1β and TNF-𝛼	LNCap (prostate cancer cell line)	[61]
Malabaricone C	Polyphenol	*Myristica malabarica* (fruits rinds)	Malabaricone C mediates the formation of ROS-dependent DNA DSB, which activate caspase-3, ATR-p38 MAPK, BAX translocation, and mitochondrial membrane potential collapse	A549 (human lung carcinoma)	[27]
Hydroxycinnamic acid derivatives (mono and dicaffeoylquinic acid)	Polyphenol	*Cynara scolymus* (artichoke head/edible part)	This polyphenol enhances cancer cell sensitivity to PTX (paclitaxel) treatment by decreasing cell proliferation both through the ROS/Nrf2 pathway (elevate ROS level) and downregulation of p-ERK that cause decrease in FEN1 expression	MCF7 and MDA-MB231 (human breast cancer)	[62]
Zerumbone	Terpenoid	*Zingiber zerumbet* rhizomes (Lempuyang)	Decreased antioxidant level and induce ROS-dependent apoptotic effect in both intrinsic and extrinsic apoptosis pathway	SW480 cell lines (human colon cancer)	[52,63,64]
Piperlongumine	Alkaloid	*Piper longum* (long pepper)	Suppresses bladder cancer cell migration/invasion mainly via ROS accumulation, Erk and PKC pathways	Human bladder cancer cell lines (T24, BIU-87, EJ)	[65]
Resveratrol	Polyphenol	*Vitis* sp. (grape skin), *Punica granatum* (pomegranate), berries, *Glycine max* (soy beans) and *Arachis hypogaea* (Peanuts)	Resveratrol act as antioxidant that can suppresses the production of ROS and inhibits COX-2 expression and prostaglandin synthesis; novel combination with salinomycin synergistically enhance the antiproliferative and proapoptotic effect by induce ROS generation	MCF-7 (human breast cancer cell)	[66]
A-terthienyl methanol	Terthiophene	*Eclipta prostrata* (false daisy)	Induce intracellular ROS and reversed NAC mechanism in arresting the S phase induces by α-terthienylmethanol	A2780, SKOV3, OVCAR3, and ES2 (human ovarian cancer cell line)	[67]
Chrysin	Polyphenol	Honey, propolis, *Passiflora caerulea* (blue passionflower)	Scavenged the ROS, inhibited proliferation (downregulation of PCNA), inflammation (downregulation of TNF-𝛼, IL-6, NF-kB, COX-2) and triggered apoptosis (upregulation of bax, caspase-9 and caspase-3).	Renal cell carcinoma	[68]
Caffeic acid	Polyphenol	*Coffea* sp. (Coffee), *Olea europaea* (olive oil), propolis	Blocks the production of ROS and suppress tumor growth and angiogenesis	Ehrlich ascites tumor	[69]
		Honey	Depleting non-protein thiol (GSH) level to elevate ROS production and decrease the mitochondrial membrane potential	Colon carcinoma cell lines HCT-15	[70]
Diallyl trisulfide	Organosulfur	*Allium* sp. (garlic)	Induce ROS increase, inhibit PI3K/Akt pathways, and induce apoptosis	Human osteosarcoma cell lines (MG-63 and MNNG/HOS)	[71]
Phenethyl isothiocyanate	Isothiocyanate	*Brassica oleracea* (broccoli, cauliflower, cabbage, kale)	Induce depletion of GSH and cause severe ROS accumulation leading to massive cell death in CCL with p53-deficiency	Chronic lymphocytic leukemia	[72]
Capsaicin	Terpenoid	*Capsicum* sp.	Inhibits the PI3K/Akt/mTOR axe to modulates autophagy by induce ROS generation and trigger ER stress	LNCaP and PC-3 (prostate cancer cell line)	[73]
Acidic pleurotus abalonus polysaccharide	Polysaccharide	*Pleurotus abalonus* (abalone mushroom)	Increase Bax/Bcl-2 ratio, caspase-9/3 activation, and poly(ADP-ribose) polymerase (PARP) degradation by increasing ROS level and cause apoptosis	MCF-7 (human breast cancer cell line)	[74]
Butein	Polyphenol	*Rhus verniciflua* Stokes	Induce ROS generation, inhibited ERK activity, enhanced p38 activation, decreased Bcl-2 expression, triggered the cleavage of pro-caspase-3 and PARP, and caused inhibited cell proliferation in cancer cells	MDA-MB-231 (human breast cancer cell line)	[75]
Gallic acid	Polyphenol	*Ceratonia siliqua* (carob fruit), *Vitis vinifera* (grape skin and seed), *Camellia sinensis* (green tea), *Fragaria vesca* (strawberries), and *Musa* sp. (bananas)	Induce ROS and intracellular Ca^2+^ production, decrease mitochondrial membrane potential level, and downregulate Bcl-2 (anti-apoptotic protein)	A375 S2 (human melanoma cell)	[76,77,78]
(−)-epicatechin	Polyphenol	*Theobroma cacao* (cocoa)	Induces ROS production and oxidative damage in cancer cells with upregulating the Bad and Bax that induce the leakage of cytochrome C into the cytoplasm and cause apoptosis	MDA-MB-231 and MCF-7 cell line (breast cancer)	[79]
Aromatic Turmerone	Polyphenol	*Curcuma longa*	Provoke ROS production that can lead to elevate ERK and JNK protein that cause increase bax/bcl-2 ratio and DR4 production. These condition cause cytochrome release and cause apoptosis	Human hepatocellular carcinoma HepG2 cells	[80]
Sulforaphane	Isothiocyanate	*Brassica oleracea* (broccoli and cauliflower)	Elevate ROS level to inhibit the recovery of hTERT expression after downregulating it and to induce ROS-dependent apoptosis in cancer cell	Human hepatocellular carcinoma Hep3B cells	[81]
Anthocyanins	Flavonoid	*Punica granatum* (pomegranate fruit extract)	Elevate the expression of phase II and antioxidant enzymes; act as ROS scavenger by activation of GSH expression; activate antioxidant response element (ARE) upstream of genes; activate Nf-kb inhibitor to induce apoptosis;	LNCaP-AR and LAPC4 cells (prostate cancer cell line)	[82,83]
Maslinic acid	Terpenoid	*Olea europaea* (olive)	Increase ROS level and induce apoptosis in cancer cell via intrinsic pathway	B16F10 melanoma cell line	[84]
